# Parameter optimization for constructing competing endogenous RNA regulatory network in glioblastoma multiforme and other cancers

**DOI:** 10.1186/1471-2164-16-S4-S1

**Published:** 2015-04-21

**Authors:** Yu-Chiao Chiu, Tzu-Hung Hsiao, Yidong Chen, Eric Y Chuang

**Affiliations:** 1 Graduate Institute of Biomedical Electronics and Bioinformatics, National Taiwan University, Taipei, Taiwan; 2 Greehey Children's Cancer Research Institute, University of Texas Health Science Center at San Antonio, San Antonio, Texas, USA; 3 Department of Medical Research, Taichung Veterans General Hospital, Taichung, Taiwan; 4 Department of Epidemiology and Biostatistics, University of Texas Health Science Center at San Antonio, San Antonio, Texas, USA; 5 Bioinformatics and Biostatistics Core, Center of Genomic Medicine, National Taiwan University, Taipei, Taiwan

## Abstract

**Background:**

In addition to direct targeting and repressing mRNAs, recent studies reported that microRNAs (miRNAs) can bridge up an alternative layer of post-transcriptional gene regulatory networks. The competing endogenous RNA (ceRNA) regulation depicts the scenario where pairs of genes (ceRNAs) sharing, fully or partially, common binding miRNAs (miRNA program) can establish coexpression through competition for a limited pool of the miRNA program. While the dynamics of ceRNA regulation among cellular conditions have been verified based on *in silico *and *in vitro *experiments, comprehensive investigation into the strength of ceRNA regulation in human datasets remains largely unexplored. Furthermore, pan-cancer analysis of ceRNA regulation, to our knowledge, has not been systematically investigated.

**Results:**

In the present study we explored optimal conditions for ceRNA regulation, investigated functions governed by ceRNA regulation, and evaluated pan-cancer effects. We started by investigating how essential factors, such as the size of miRNA programs, the number of miRNA program binding sites, and expression levels of miRNA programs and ceRNAs affect the ceRNA regulation capacity in tumors derived from glioblastoma multiforme patients captured by The Cancer Genome Atlas (TCGA). We demonstrated that increased numbers of common targeting miRNAs as well as the abundance of binding sites enhance ceRNA regulation and strengthen coexpression of ceRNA pairs. Also, our investigation revealed that the strength of ceRNA regulation is dependent on expression levels of both miRNA programs and ceRNAs. Through functional annotation analysis, our results indicated that ceRNA regulation is highly associated with essential cellular functions and diseases including cancer. Furthermore, the highly intertwined ceRNA regulatory relationship enables constitutive and effective intra-function regulation of genes in diverse types of cancer.

**Conclusions:**

Using gene and microRNA expression datasets from TCGA, we successfully quantified the optimal conditions for ceRNA regulation, which hinge on four essential parameters of ceRNAs. Our analysis suggests optimized ceRNA regulation is related to disease pathways and essential cellular functions. Furthermore, although the strength of ceRNA regulation is dynamic among cancers, its governing functions are stably maintained. The findings of this report contribute to better understanding of ceRNA dynamics and its crucial roles in cancers.

## Background

A group of short single-stranded RNAs, namely microRNAs (miRNAs), has been widely investigated in this decade. With an average length of 22 nucleotides only, miRNAs are not protein coding transcripts. Instead, they fulfill the role of regulators of gene expression by complementarily binding to 3' untranslated regions (3' UTRs) of target mRNA transcripts [1,2]. According to existing biological evidence, the binding of miRNAs on mRNA can cause mRNA degradation or suppression of translation, and may affect expression of up to one third of the protein coding genes in humans [2]. In cancers, the dysregulation of miRNAs has been proven to be involved in oncogenesis (reviewed in [3]), tumor progression [4,5], and clinical outcomes, such as patient survival [6,7]. With advances in next-generation sequencing, a great number of novel miRNAs have been identified and deposited in the public database miRBase [8], increasing the complexity of miRNA regulation.

Recently, reports postulated and experimentally validated that miRNAs can serve as an alternative layer of post-transcriptional gene-gene regulation, namely the competing endogenous RNAs (ceRNAs) [9-11]. Pairs of genes (ceRNAs) fully or partially sharing common binding miRNAs can establish crosstalk with each other through competition for a limited pool of the common miRNAs (miRNA programs; abbreviated as miRP). When expression level of one ceRNA rises (or decreases) in cells, it attracts (or releases) the targeting miRNAs away from (or toward) the other ceRNAs, and in turn has protective (or degradative) effects on expression of the other ceRNA partners. In other words, this postulation provides the scenario that genes can, facilitated by miRNAs, regulate each other without direct interaction. Through bioinformatic analysis and *in vitro *experiments on the tumor suppressor gene *PTEN*, previous studies suggested that ceRNAs of *PTEN*, *e.g*., *VAPA *and *ZEB2*, can possess tumor-suppressive properties by modulating (*i.e*. coexpressing with) *PTEN *expression levels in a miRNA-dependent while protein-coding independent manner [9-11]. Through *in silico *analysis of glioblastoma gene expression datasets, our recent study further demonstrated that ceRNA regulation, while only accounting for a small portion of global gene regulation, plays an essential role in transient cellular responses to dynamic inter-cellular signals [12]. Taken together, these observations have revealed that ceRNA regulation provides an alternative mechanism of gene regulation in essential cellular processes and functions. To address the optimal cellular conditions for ceRNA regulation, several recent studies used the mathematical mass-action simulation [13,14] and cell line experiments [13] to demonstrate the dependency of ceRNA regulation on the dosages (*i.e*., cellular concentration) of both ceRNAs and miRPs, and number of miRNA response elements, suggesting the dynamic and condition-specific properties of ceRNA regulation *in vitro*.

Realizing that biological processes typically involve more complex mechanisms *in vivo *than *in vitro*, in the first part of this study we investigate the optimal conditions of ceRNA regulation in expression datasets derived from clinical samples. The optimal conditions may depend on the following essential factors: 1) Size of miRNA programs, 2) Number of miRNA program binding sites, 3) Expression level of miRNA programs, and 4) Expression level of ceRNAs. Here we developed an analytic scheme for determining whether these factors affect strength of ceRNA regulation. By integrating four factors' effects, the biological functions governed by optimal ceRNA regulation can be elucidated. On the other hand, while pan-cancer genomic analysis has been widely utilized to reveal tumor-specific and distinct molecular signature to better understand cancer heterogeneity [15,16], pan-cancer analysis of ceRNA regulation, to our knowledge, has not been systematically explored. Collectively, the present study provides a systematic investigation of optimal conditions for ceRNA regulation, explores associated biological functions, and conducts pan-cancer analysis of ceRNAs in four cancer types.

## Results

### Model overview and data preparation

**Figure 1:**
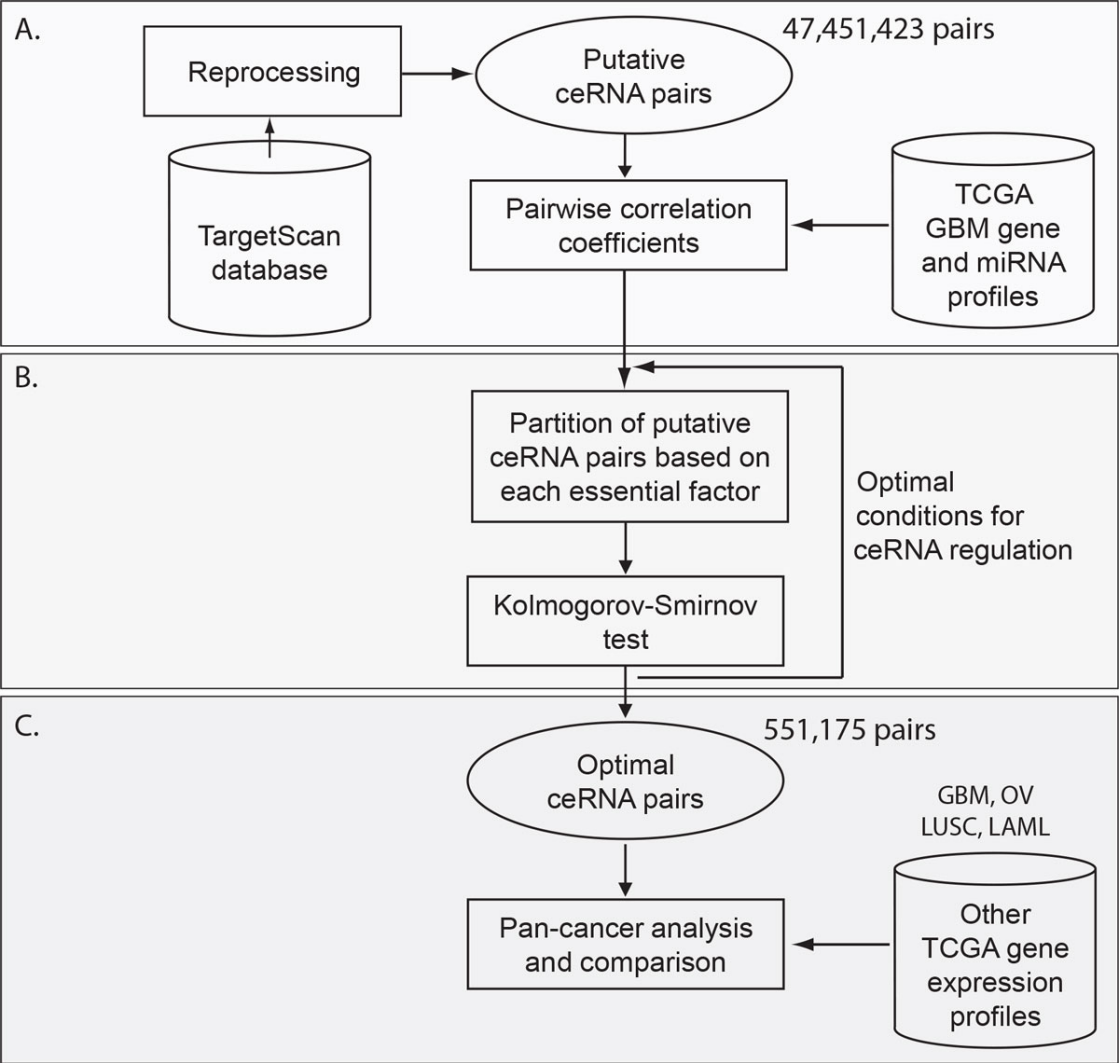
**Analysis flowchart of this study**. The present study is aimed to systematically explore optimal conditions and related biological functions of ceRNA regulation in GBM, and confer cancer type specific and independent effects. (**A**) First we defined 47,451,423 putative ceRNA pairs as pairs of genes (*i.e*., pairs of ceRNAs) sharing any number of predicted targeting miRNAs in the TargetScan database. Pairwise correlation coefficients of putative ceRNA pairs were computed in 481-sample TCGA GBM datasets. (**B**) Correlation coefficients were partitioned into groups based on the states of each essential factor of putative ceRNA pairs, followed by inter-group goodness-of-fit tests (K-S tests) that pinpointed the essential factors and optimal conditions for ceRNA regulation. (**C**) 551,175 pairs of ceRNAs fulfilling all of the identified optimal conditions were defined as optimal ceRNA pairs. In order to address differential and constitutive functions, we then included TCGA OV, LUSC, and LAML datasets and performed pan-cancer analysis.

The proposed study aims to comprehensively explore optimal conditions for ceRNA regulation, to investigate functions governed by ceRNA regulation, and to evaluate pan-cancer effects. We started by investigating how essential factors, such as the size of miRNA programs, the number of miRNA program binding sites, and expression levels of miRNA programs and ceRNAs affect the ceRNA regulation in tumor samples from glioblastoma multiforme (GBM) patients. Here we chose GBM as the model cancer type because it is one of the most frequently studied cancers in investigating ceRNA regulation [10,11]. The analysis flowchart is illustrated in Figure 1. We analyzed 481 tumor sets with tumor-matched mRNA and miRNA expression profiles from TCGA [17]. Based on the definition of previous studies, we defined putative ceRNA pairs as two genes sharing any number of common predicted targeting miRNAs. Recruiting the prediction data from the TargetScan algorithm [2,18], we identified 47,451,423 putative ceRNA pairs with least one common targeting miRNA, comprising 10,872 ceRNAs (genes). For each of the putative ceRNA pairs, the pairwise correlation coefficient of gene expression profiles was computed (Figure 1A). Varying size of miRNA program, for example, generated multiple cumulative distribution functions (CDFs) of correlation coefficients. We then performed the goodness-of-fit tests (Kolmogorov-Smirnov test; K-S test) among the CDFs to pinpoint whether or not the optimal conditions for intensified ceRNA regulation depend on the essential factors (Figure 1B). Here the intensified ceRNA regulation refers to the overall increased degree of coexpression of ceRNA pairs. Upon identification of optimal conditions with respect to the four essential factors, we defined the optimal ceRNA pairs from the putative ceRNAs, which satisfy all the four optimal conditions. We then performed functional annotation analysis to investigate biological processes and functions governed by optimal ceRNA regulation (Figure 1C). To further address the cancer type-specific and independent effects, we evaluated pairwise coexpression of the optimal ceRNA pairs identified from GBM in other TCGA cancer datasets, including 585-sample ovarian serous cystadenocarcinoma (OV) [19], 133-patient lung squamous cell carcinoma (LUSC) [20], and 197-sample acute myeloid leukemia (LAML) [21]. The comprehensive results from the functional annotation level and from the enrichment of signaling pathways were reported in later sections below.

### Increased size of miRNA program and number of miRNA program binding sites intensify ceRNA regulation in GBM

**Figure 2:**
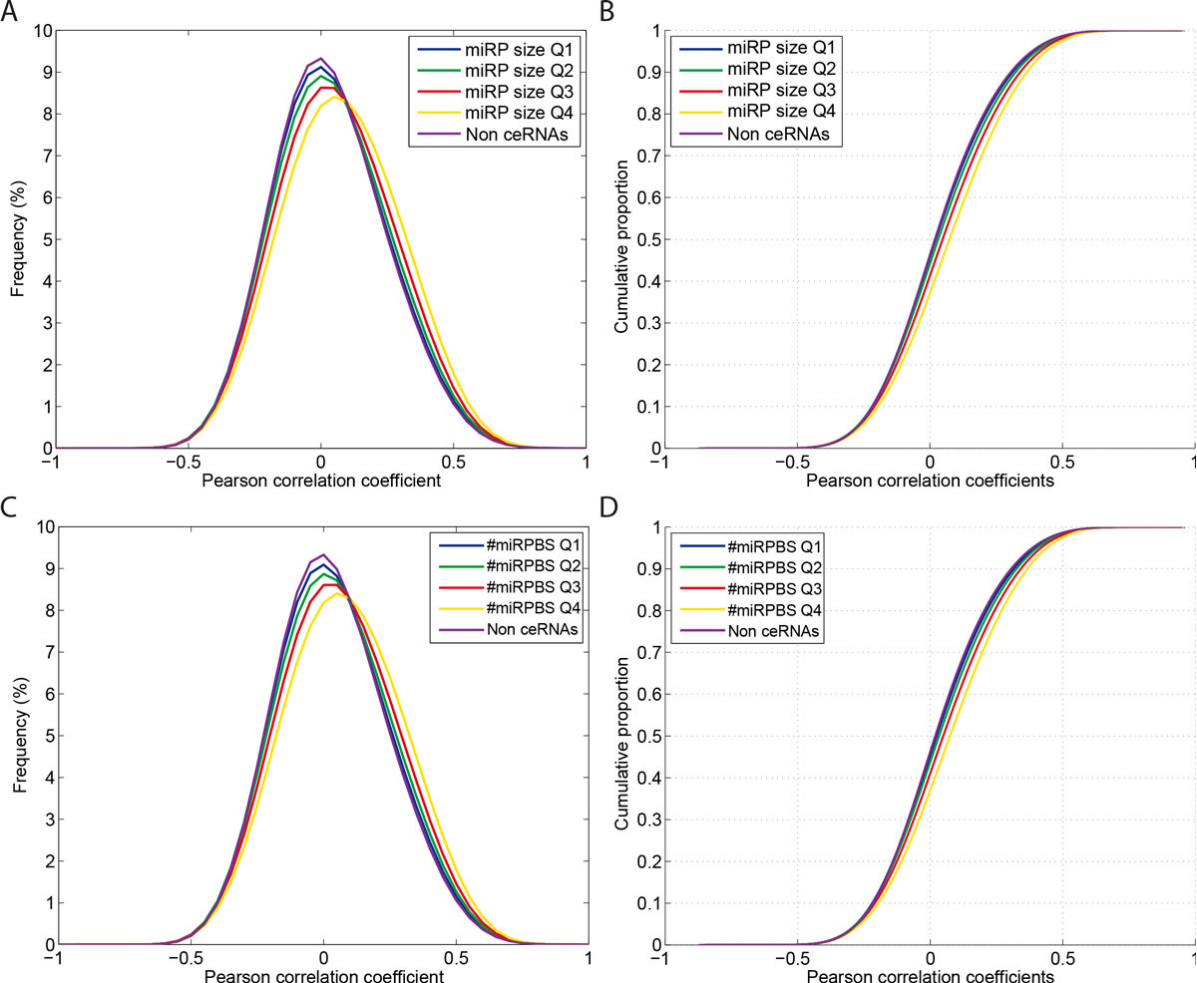
**Effects of size of miRNA programs and number of miRNA program binding sites on ceRNA regulation**. **(A, B) **Density functions and cumulative distribution functions of correlation coefficients of putative ceRNA pairs. The putative ceRNA pairs were divided into four groups by the quartiles of miRNA program sizes. **(C, D) **Density functions and cumulative distribution functions of correlation coefficients of putative ceRNA pairs, which were partitioned based on number of miRNA program binding sites.

The size of miRNA programs (number of common targeting miRNAs) among the 47,451,423 putative ceRNA pairs ranged from 1 to 262, with quartiles of 2, 6, and 14 (Additional file 1: Figure S1). In order to dissect the association of size of miRPs with ceRNA regulation, we divided the putative ceRNA pairs into 4 groups based on size of miRPs, (i) 7,382,212 putative pairs < 25^th^-percentile, (ii) 15,289,167 pairs within 25^th^- 50^th ^percentiles, (iii) 12,163,761 pairs within 50^th^-75^th ^percentiles, and (iv) 12,616,283 pairs ≥ 75^th^-percentile, and compared the distributions of correlation coefficients across groups. Coexpression of ceRNA pairs was significantly elevated with increasing size of miRPs (K-S test *p*-value < computing precision of double-precision floating point, hereafter referred to as *p*-value ~0, between any two groups) while correlation coefficients of 13,043,077 non-ceRNA pairs followed approximately the null random distribution (Figure 2A-B).

As defined in the Methods section, the number of miRNA program binding sites (#miRPBS) was determined by summing up the total number of interacting sites of miRPs on the corresponding pairs of ceRNAs. Among all the putative ceRNA pairs the #miRPBS fell in the range of 2-1,859 (histogram in Additional file 2: Figure S2). We grouped the putative ceRNA pairs based on the #miRPBS with identical criteria as used in analyzing size of miRNA programs, resulting in 4 groups of 10,829,459, 12,443,877, 12,296,702, and 11,881,385 putative ceRNA pairs. With the K-S tests, significant *p*-values between any two groups (*p*-value ~0) indicate that the number of miRP binding sites is positively associated with ceRNA coexpression (Figure 2C-D). Taken together, our data demonstrate that increased numbers of common targeting miRNAs as well as the abundance of binding sites intensify the strength of ceRNA regulation.

### Strength of ceRNA regulation is dependent on expression levels of miRNA programs and ceRNAs in GBM

**Figure 3:**
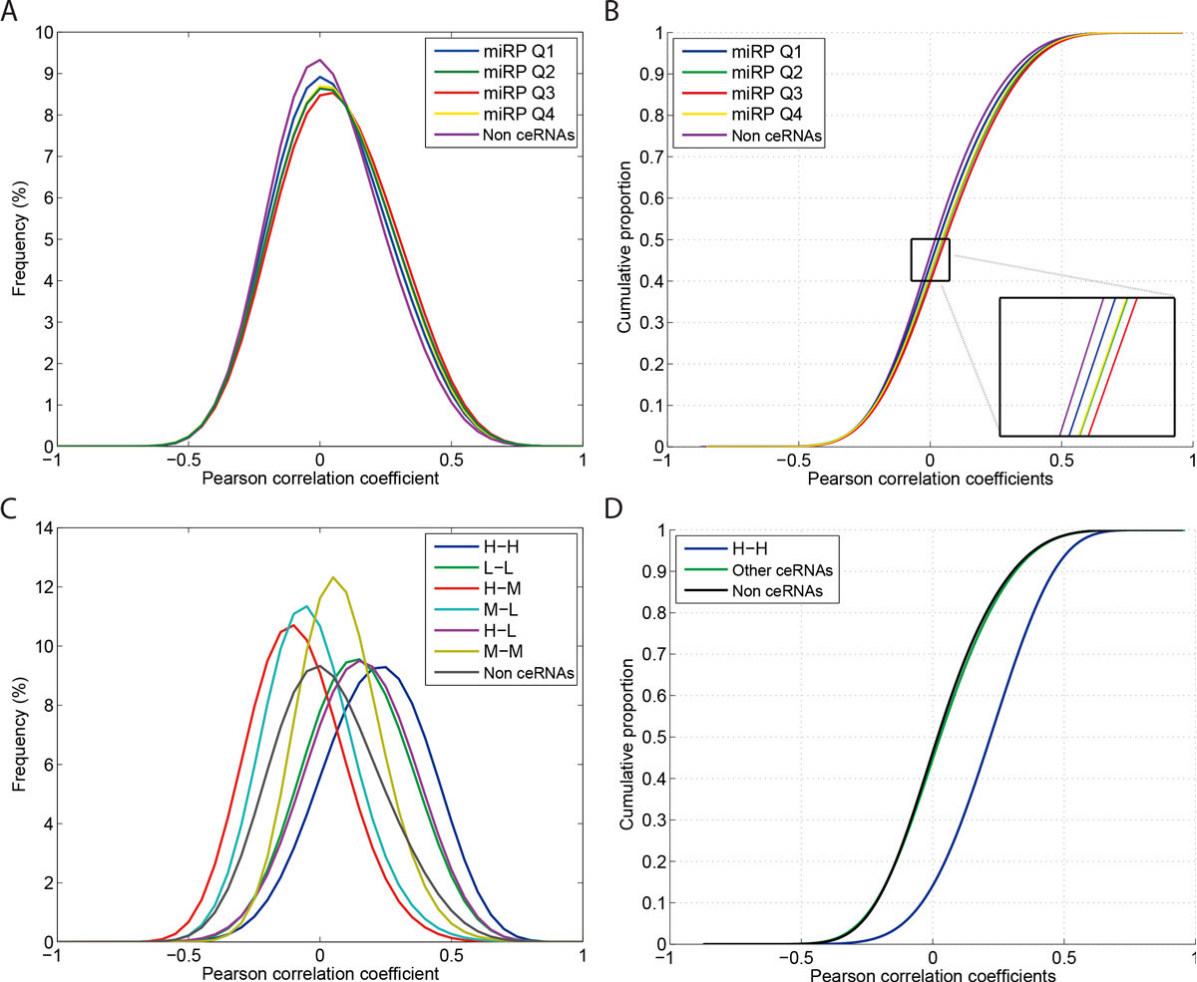
**Effects of expression levels of miRNA programs and ceRNAs on ceRNA regulation**. **(A, B) **Density functions and cumulative distribution functions of correlation coefficients of putative ceRNA pairs. The putative ceRNA pairs were split into groups by quartiles of miRNA programs expression levels. **(C) **Density functions of correlation coefficients of putative ceRNA pairs. Here the putative ceRNA pairs were categorized based on expression states (*i.e*., H, M, and L) of their composed ceRNAs. **(D) **Cumulative distribution functions of **(C)**, focused on comparison of ceRNA pairs composed of two highly expressed genes (H-H) to other ceRNA pairs and non-ceRNA pairs.

We reasoned that ceRNA regulation might depend on expression levels of miRNAs in miRPs. To test this hypothesis, we split the putative ceRNAs into 4 groups by the quartiles of expression levels of miRPs (6.29, 6.88, and 7.52 in log2 scale; histogram in Additional file 3: Figure S3), resulting in 11,862,855 ceRNA pairs in each group. Here the expression levels of miRPs were calculated by simply averaging the expression levels of miRNAs in miRPs for each ceRNA pair. All of the four groups (*i.e*., Q1 to Q4) showed significantly different distributions of correlation coefficients from non-ceRNA pairs and the Q3 group showed the most right-shifted distribution function (Figure 3A-B). Among the four groups, remarkably, the putative ceRNA pairs with median miRP expression levels (Q2 and Q3) exhibited higher correlation than ones belonging to Q1 and Q4 (K-S test *p*-value ~0). This result was in agreement with the common assumption that both excessive abundance (loss of competition) and sparse availability (nothing to compete for) of miRNA transcripts will reduce regulation.

We further investigated whether ceRNA expression levels play a crucial role in governing ceRNA regulation. 3,653, 3,222, and 3,343 genes with mean expression levels across the samples falling in the range of 5-35, 35-65, and 65-95 percentiles of all 10,872 genes analyzed, respectively, were annotated as low (L), medium (M), and high (H) expression states. We note that in the analysis we eliminated the genes within the bottom or top 5% expression levels, which may be largely attributed to background or saturation noise. Putative ceRNA pairs were then categorized based on expression states of their composed ceRNAs (see Table 1 for number of ceRNA pairs in each group). Interestingly, the results revealed high involvement of expression levels of ceRNAs in determining ceRNA regulation (Figure 3C). 4,551,383 (9.59%) putative ceRNA pairs composed of two highly expressed genes showed significant coexpression (H-H) compared to other ceRNA pairs and non-ceRNA pairs (K-S statistics = 0.356 and 0.378 and both K-S test *p*-values ~0; Figure 3D). Overall, the results suggest that ceRNA regulation is dependent on expression levels of both miRPs and ceRNAs. Remarkably, our observation from analysis of clinical microarray data agrees with Ala *et al*.'s data using a mathematical mass-action model in that optimal cellular conditions for ceRNA regulation depend on expression levels of miRNA programs as well as ceRNAs [13].

**Table 1:** Number of ceRNA pairs in groups categorized based on expression states of their composed ceRNAs.

	High-expression genes (H)	Medium-expression genes (M)	Low-expression genes (L)
**High-expression genes (H)**	4551383 (9.59%)		

**Medium-expression genes (M)**	8262660 (17.41%)	3786914 (7.98%)	

**Low-expression genes (L)**	10134010 (21.36%)	9302868 (19.61%)	5654651 (11.92%)

The percentages were calculated with respect to the number of all putative ceRNA pairs (47,451,423).

### Intertwined signaling among optimal ceRNAs is associated with essential cellular functions and disease pathways

**Figure 4:**
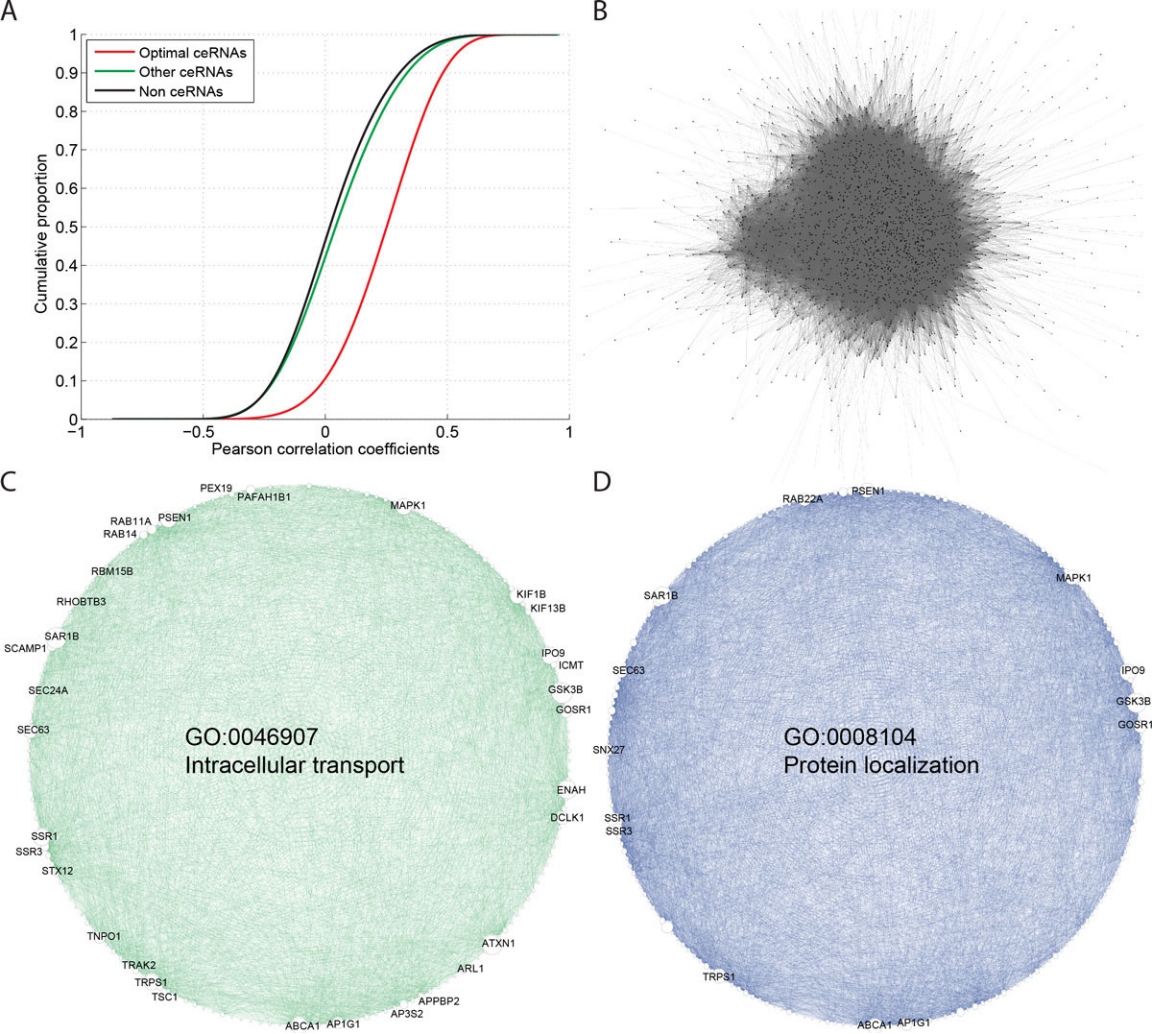
**The optimal ceRNA regulatory network**. **(A) **Cumulative distribution functions of correlation coefficients of optimal ceRNA pairs satisfying four optimal conditions, other ceRNA pairs, and non-ceRNA pairs. **(B) **The optimal ceRNA regulatory network. The network is constructed by merging the identified 551,175 optimal ceRNA pairs comprising 2,405 ceRNAs. Nodes and edges denote ceRNAs and optimal regulatory relationship, respectively. **(C) **The subnetwork of intracellular transport (GO:0046907), generated by extracting 181 genes related to the function and corresponding ceRNA regulatory pairs from **(B)**. Node size is proportional to the number of first-order neighbors and nodes accounting for more than 1% of all intra-function ceRNA regulating pairs are labeled with gene symbols. **(D) **The subnetwork of protein localization (GO:0008104).

Observing that regulation strength is intensified in ceRNA pairs with a large size of miRP (Q4), large number of miRP binding sites (Q4), appropriate miRP expression levels (Q3), and high expression levels of both partner ceRNAs (H-H), we then defined 551,175 pairs of ceRNAs satisfying all of the four optimal conditions as optimal ceRNA pairs. The optimal ceRNA pairs accounted for only 1.16% of all putative ceRNA pairs, containing 2,405 optimal ceRNAs (22.12%) of all 10,872 ceRNA genes. Additional file 4: Table S1 includes list of the optimal ceRNA pairs and summary of optimal ceRNAs. Pairwise coexpression of the optimal ceRNA pairs led other ceRNA pairs and non-ceRNAs by large margins (K-S statistics = 0.391 and 0.442 and both K-S test *p*-values ~0, Figure 4A). In order to dissect higher-order properties of inter-ceRNA signaling, we merged the identified optimal ceRNA pairs and constructed the optimal ceRNA regulatory network (Figure 4B). On average each ceRNA directly interacted with up to ~458 optimal ceRNA partners, suggestive of the complex signaling maintained by ceRNA regulation. Connected to 1,480 first-order neighbors *CDS2 *(CDP-diacylglycerol synthase (phosphatidate cytidylyltransferase) 2) was found as the top hub ceRNA in the network, with the greatest number of first-order neighbors. Many of the top 20 hub genes were previously reported to be associated with cancer (8 genes), neurological diseases (9), hereditary disorders (9), or the function of cell death and survival (7) (data from database of Ingenuity Pathway Analysis (Qiagen Inc.), Table 2). Notably, the well-studied prognosis biomarker in cancers, SMAD family member 4 (*SMAD4*), regulated up to 1,382 optimal ceRNAs and was ranked 12^th ^in the hub ceRNA list. Functional annotation analysis indicated that the 2,405 optimal ceRNAs played crucial roles in the biological functions of intracellular transport (GO:0046907, Bonferroni adjusted *p*-value = 9.26 × 10-18) and protein localization (GO:0008104, Bonferroni adjusted *p*-value = 7.88 × 10-17). Figure 4C-D depicts complex intra-function ceRNA regulation within the two functions. For a more comprehensive overview of functional annotation, we utilized The Database for Annotation, Visualization and Integrated Discovery (DAVID) [22,23] to analyze the enriched clusters of Gene Ontology (GO) biological process and molecular function terms. The top five clusters of functions were protein transport, protein catabolic process, vehicle-mediated transport, protein modification/ubiquitination, and regulation of translation (see Table 3 DAVID cluster scores > 5). Taken together, our data indicate that optimal ceRNA regulation is highly involved in diseases and maintenance of essential cellular functions.

**Table 2:** Top 20 hub genes of the optimal ceRNA network.

Hub genes	No. of first-order neighbors	Percentage of total optimal ceRNA pairs	Entrez gene name	Type^a^	Disease/Function^a^
CDS2	1480	0.269%	CDP-diacylglycerol synthase (phosphatidate cytidylyltransferase) 2	enzyme	

PARVA	1470	0.267%	parvin, alpha	other	cancer; cell death and survival

SLC1A2	1466	0.266%	solute carrier family 1 (glial high affinity glutamate transporter), member 2	transporter	neurological disease; hereditary disorder

NFIB	1435	0.260%	nuclear factor I/B	transcription regulator	

SSR1	1421	0.258%	signal sequence receptor, alpha	other	

GTF2H5	1407	0.255%	general transcription factor IIH, polypeptide 5	other	neurological disease; hereditary disorder

SAR1B	1398	0.254%	SAR1 homolog B (S. cerevisiae)	enzyme	hereditary disorder

GSK3B	1392	0.253%	glycogen synthase kinase 3 beta	kinase	cancer; cell death and survival; neurological disease; hereditary disorder

HEG1	1390	0.252%	heart development protein with EGF-like domains 1	other	cancer

ZNF148	1390	0.252%	zinc finger protein 148	transcription regulator	cell death and survival

EIF5	1387	0.252%	eukaryotic translation initiation factor 5	translation regulator	cancer

SMAD4	1382	0.251%	SMAD family member 4	transcription regulator	cancer; cell death and survival; prognosis biomarker; neurological disease; hereditary disorder

TCF4	1374	0.249%	transcription factor 4	transcription regulator	cell death and survival; neurological disease; hereditary disorder

QKI	1366	0.248%	QKI, KH domain containing, RNA binding	other	cancer; neurological disease; hereditary disorder

LSAMP	1364	0.247%	limbic system-associated membrane protein	other	

ATXN1	1354	0.246%	ataxin 1	transcription regulator	cancer; cell death and survival; neurological disease; hereditary disorder

DCP2	1354	0.246%	decapping mRNA 2	enzyme	

PSD3	1346	0.244%	pleckstrin and Sec7 domain containing 3	other	cancer; neurological disease

SLC38A1	1340	0.243%	solute carrier family 38, member 1	transporter	

VAPB	1340	0.243%	VAMP (vesicle-associated membrane protein)- associated protein B and C	other	cell death and survival; neurological disease; hereditary disorder

^a ^Annotations were obtained from Ingenuity Pathway Analysis (Qiagen Inc.).

**Table 3:** Top 5 clusters of Gene Ontology terms enriched in the 2,405 optimal ceRNAs

GO Term	No. of genes	Bonferroni adjusted *P*-value	Total No. of optimal ceRNA pairs/ceRNAs	GBM core^a^	OV core^a^	LUSC core^a^	LAML core^a^	CV among cancers^b^
**Cluster 1 (Enrichment Score: 17.99)**								

GO:0046907~intracellular transport	184	9.26E-18						
						
GO:0008104~protein localization	225	7.88E-17						
						
GO:0015031~protein transport	200	4.67E-16						
						
GO:0045184~establishment of protein localization	201	6.18E-16	8229/261	7152/261	6235/258	3961/254	2755/247	34.85%/2.06%
						
GO:0070727~cellular macromolecule localization	126	2.75E-14						
						
GO:0034613~cellular protein localization	124	1.09E-13						
						
GO:0006886~intracellular protein transport	111	3.17E-11						

**Cluster 2 (Enrichment Score: 8.27)**								

GO:0009057~macromolecule catabolic process	180	2.95E-08						
						
GO:0044265~cellular macromolecule catabolic process	168	1.04E-07						
						
GO:0030163~protein catabolic process	143	6.75E-06						
						
GO:0043632~modification-dependent macromolecule catabolic process	134	8.41E-06						
						
GO:0019941~modification-dependent protein catabolic process	134	8.41E-06	4838/200	4418/200	3836/197	2379/193	2049/191	31.08%/1.79%
						
GO:0051603~proteolysis involved in cellular protein catabolic process	138	1.31E-05						
						
GO:0044257~cellular protein catabolic process	138	1.85E-05						
						
GO:0006511~ubiquitin-dependent protein catabolic process	68	8.96E-05						
						
GO:0006508~proteolysis	177	1						

**Cluster 3 (Enrichment Score: 6.01)**								

GO:0016192~vesicle-mediated transport	141	8.98E-08						
						
GO:0016044~membraneorganization	93	3.70E-04	3462/157	2882/155	2519/156	1607/149	1208/147	32.82%/2.53%
						
GO:0010324~membrane invagination	50	0.935246						
						
GO:0006897~endocytosis	50	0.935246						

**Cluster 4 (Enrichment Score: 5.67)**								

GO:0032446~protein modification by small protein conjugation	45	6.85E-05						
						
GO:0070647~protein modification by small protein conjugation or removal	50	2.36E-04						
						
GO:0016567~protein ubiquitination	40	7.30E-04						
						
GO:0019787~small conjugating protein ligase activity	45	0.012039	607/67	555/67	488/66	335/65	266/62	28.13%/2.88%
						
GO:0016881~acid-amino acid ligase activity	51	0.020482						
						
GO:0004842~ubiquitin-protein ligase activity	39	0.084550						
						
GO:0016879~ligase activity, forming carbon-nitrogen bonds	53	0.213866						

**Cluster 5 (Enrichment Score: 5.10)**								

GO:0010608~posttranscription al regulation of gene expression	59	0.001017						
						
GO:0006417~regulation of translation	40	0.036298	1312/105	1132/105	961/101	643/99	480/100	31.90%/2.25%
						
GO:0032268~regulation of cellular protein metabolic process	96	0.576893						

^a ^Number of optimal ceRNA pairs/ceRNAs with significant positive correlation coefficients in corresponding cancer dataset, ^b ^Coefficients of variation of number of core ceRNA pairs/ceRNAs among four cancer datasets.

### Pan-cancer analysis revealed dynamic ceRNA regulation among constitutive ceRNAs

**Figure 5:**
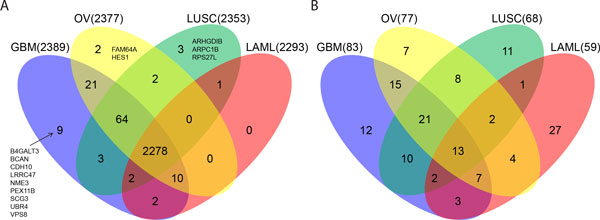
**Venn diagrams of core ceRNAs among four cancer types**. **(A) **Comparison of core ceRNAs in four cancer datasets. Core ceRNAs are genes that comprise the core ceRNA pairs with significant positive correlation coefficients in a cancer dataset. **(B) **Comparison of top hub core ceRNAs which collectively account for 10% of the total core ceRNA regulating pairs in corresponding cancer datasets.

For further conferring similarity/dissimilarity of ceRNA regulation and functions among different cancer types, we analyzed pairwise correlation coefficients of the 551,175 optimal ceRNA pairs in TCGA datasets of GBM, OV, LUSC, and LAML. Among the optimal ceRNA pairs, 452,718 (82.14%), 358,359 (65.02%), 239,886 (43.52%), and 106,940 (19.40%) pairs (hereafter referred to as core ceRNA pairs) exhibited significantly positive correlation coefficients (right-tail *p*-value < 0.05) in GBM, OV, LUSC, and LAML, respectively. Notably, although numbers of significant regulating pairs changed immensely, the involved ceRNAs were largely similar among cancer types: 2,389 (99.33% out of 2,405 optimal ceRNAs), 2,377 (98.84%), 2,353 (97.84%), and 2,293 (95.34%) ceRNAs (core ceRNAs) in four cancer types, with 2,278 in common (Figure 5A). For the most highly connected core ceRNAs in each cancer, LAML showed relatively distinctive results from the solid tumors, with 27 out of the 59 (45.76%) LAML hub core ceRNAs appearing exclusively in LAML (Figure 5B). Detailed lists of core ceRNA pairs are tabulated in Additional file 5: Table S2. Also, the four sets of core ceRNAs shared highly identical enriched biological functions with the optimal ceRNAs (data not shown). Among the five clusters of GO terms associated with the optimal ceRNAs, the number of intra-function core ceRNA pairs varied massively (coefficients of variation all > 28.13%, Table 3) while the number of comprising ceRNAs remained relatively stable (coefficients of variation ranging from 1.79% to 2.88%, Table 3). The top (protein transport) and the 4^th ^(protein modification/ubiquitination) clusters were found with the largest changes in number of core ceRNA pairs and ceRNAs, respectively (see Additional file 6: Figure S4 and Additional file 7: Figure S5 for network visualization). Furthermore, our analyses revealed that, although massive re-wiring underlies ceRNA regulation among cancer types, the overall topology (recruitment of ceRNAs) of the core ceRNA networks was relatively stable, maintained mainly by a tiny subset of ceRNA regulating pairs with an extremely high degree of coexpression (Additional file 8: Figure S6). Incorporating these observations, we concluded that while the strength of ceRNA regulation is dynamic across cancer types, the essential biological functions governed by ceRNA regulation are stably retained.

## Discussion

Besides the well-studied role of miRNAs in directly regulating gene expression, emerging evidence postulates that ceRNA regulation is an alternative mechanism through which miRNAs participate in gene regulation. Regulation of ceRNAs has been proved to govern essential biological functions in human development and diseases including cancer (reviewed in [24,25]). While recent studies have proved the dynamicity of ceRNA regulation among cellular conditions based on *in silico *and *in vitro *experiments [13,14], comprehensive investigation into the strength of *in vivo *ceRNA regulation remains largely unexplored. Addressing this, in the present study we started by characterizing crucial factors in determination of optimal conditions using gene expression profiles derived from tumor specimens from TCGA. Our analyses indicated the dose effect of miRNA programs; *i.e*., increased size of miRNA programs as well as increased number of miRNA program binding sites enhance the competing relationship among genes and thus elevate inter-ceRNA coexpression. Furthermore, the expressional levels of both miRNA programs and ceRNAs affect ceRNA regulation and lead to statistically significant differences in distributions of correlation coefficients, suggestive of the existence of optimal molecular conditions in which ceRNA regulation prevails. Intermediate expression levels of miRNA programs allow efficient and effective competition, thus further optimize the power of ceRNA regulation; while varied expression levels of ceRNAs exhibited divergent effects on ceRNA regulation. Taken together, our analyses clearly demonstrated that ceRNA regulation highly depends on states of the essential factors and thus may involve complex and dynamic processes *in vivo*. Incorporating the optimal conditions of ceRNA regulation, we identified the optimal ceRNA pairs and revealed the biological functions significantly associated with protein transport, protein catabolic processes, and regulation of translation. These functions are all of essential significance in regular cellular routines, indicative of the indispensable involvement of ceRNA regulation *in vivo*. Recently, Denzler *et al*. assessed the ceRNA effect in hepatocytes and liver using quantitative biological experiments [26]. Our findings agree with their paper where it was concluded that ceRNA regulation is more likely to occur when both ceRNAs are highly expressed or miRNA binding sites are sufficient. Interestingly, analyzing the unusually highly expressed miRNA miR-122, they showed that coexpression of miR-122 target genes was achieved specifically at extremely high target site abundance. Our data further showed the dependence of ceRNA regulation on the essential factors and cancer types. Taken together, we elucidated that ceRNA regulation is a complex and sophisticated mechanism *in vivo*, thus difficult to be observed under some cellular conditions. Future biological studies may investigate it in detail and carry out essential clues to complex ceRNA regulation.

With the growing volume of DNA microarray and next-generation sequencing samples, pan-cancer analysis has unveiled both common and unique characteristics of genomic aberrations [15], expression profiles [27], oncogenic microRNAs [16], and secretome [28] across cancer types. This emerging research domain illuminates the tumor type-specific and independent molecular properties, and further contributes to enhanced understanding of tumorigenesis and progression. In this pioneer report, for better characterizing ceRNA in cancers, we extended the optimal ceRNA pairs identified from GBM to large datasets deposited in TCGA, including ovarian serous cystadenocarcinoma, lung squamous cell carcinoma, and acute myeloid leukemia datasets. Our results demonstrated that ceRNA regulatory networks are massively rewired across cancer types. Acute myeloid leukemia exhibited the most distinctive ceRNA pattern from other solid tumors. Remarkably, although the strength and wiring of ceRNA regulation changed immensely, the recruitment of genes into the ceRNA regulatory network is highly stable among cancer types; *i.e*., the highly intertwined ceRNA regulatory relationship enables genes to be effectively regulated by some of their ceRNA partners regardless of perturbations to cellular conditions. This property of ceRNA regulation stabilizes intra-function regulation and thus facilitates maintenance of essential biological functions in cells. With the increasing number of molecular profiles of cancers, future analysis may extend the analysis to more cancer types and provide universal landscape of ceRNA regulation.

In the present study, for each of four factors we attempted with the quartiles and specific percentiles to partition the putative ceRNAs into groups and compare inter-group distribution of correlation coefficients. For inferring more subtle changes in distribution of correlation coefficients, future work may use other methods that are capable of revealing local fluctuations of distribution functions. For defining putative ceRNA pairs, we employed prediction data from the miRNA-target gene prediction algorithm of TargetScan. TargetScan is a widely used prediction algorithm that takes into consideration both sequence complementarity (especially the seed regions of miRNAs) and conservativity of binding sites. There are still a handful of prediction algorithms, such as PicTar, based on genome-wide sequence alignment [29] and mirBridge utilizing gene set enrichment analysis [30]. While different prediction methods, as well as species-specific targeting, define dissimilar miRNA-target gene pairs, since our present report was aimed to investigate the systematic view of ceRNA regulation and its optimal conditions, out of simplicity we only employed the TargetScan algorithm. Indeed, ceRNAs with larger number of targeting miRNAs are expected to have more putative ceRNA partners, and thus account for a higher proportion of the 47 million putative ceRNA pairs. However, in the analysis of optimal conditions for each parameter, the calculation of correlation coefficients was based on "ceRNA pairs" instead of "ceRNAs". Furthermore, since TargetScan is one of the algorithms with the highest prediction precision (reviewed in [31]), in this study the putative ceRNA pairs were defined with high confidence, regardless of the numbers of their ceRNA partners. Thus, we reason that differences in the number of ceRNA partners among genes would not cause major systematic biases to our analysis.

Besides, here we adopted the biologically straightforward Pearson correlation as a measure of gene-gene coexpression, other methods such as mutual information and polynomial regression may provide alternatives for modeling non-linear properties of miRP-modulated coexpression of ceRNAs. In the report, out of simplicity we focused on "pairwise" relationship between ceRNA pairs. However, the competition for a set of miRNAs may not be exclusively limited to pairs of ceRNAs since a handful of ceRNAs can compete, fully or partially, for common targeting miRNAs. Realizing that one miRNA can target up to hundreds of mRNAs in the genome-wide scale, taking all these factors into account will exponentially complicate the problem and thus require more complex mathematical models.

## Conclusions

Here we carried out a comprehensive investigation into optimal conditions for competing endogenous RNA regulation, associated biological functions, and pan-cancer effects of ceRNA regulation. Using TCGA GBM microarray datasets, we demonstrated that regulation between ceRNAs is dynamic, however the optimal conditions are quantifiable. The obtained optimal ceRNA regulatory network is associated with diseases pathways and essential cellular functions. Pan-cancer analysis revealed that while strength of ceRNA regulation is dynamic across cancer types, the highly intertwined ceRNA signaling stably maintains the essential functions it governs. Therefore, we expect the study presented here brings biological insights into the dynamicity and essential roles of ceRNA regulation.

## Methods

### Microarray expression datasets

Microarray datasets of glioblastoma multiforme (GBM) [17], ovarian serous cystadenocarcinoma (OV) [19], lung squamous cell carcinoma (LUSC) [20], and acute myeloid leukemia (LAML) [21] patients were downloaded from The Cancer Genome Atlas (TCGA) database. We extracted 481 GBM samples with paired miRNA and mRNA expression profiles from the datasets of 557-sample Affymetrix Human Genome U133A Arrays and 505-sample Agilent 8 × 15K Human miRNA-specific microarrays. The OV, LUSC, and LAML datasets were composed of 585-sample Affymetrix Human Genome U133A Arrays, 133-sample Affymetrix Human Genome U133A Arrays, and 197-sample Affymetrix Human Genome U133 Plus 2.0 Arrays, respectively. We utilized TCGA Level 3 data, which were previously normalized and merged into gene- or miRNA-level expression values by TCGA, for consequent analysis in this study.

### MiRNA targeting genes

For defining miRNA targeting genes we adopted miRNA-target gene prediction data from the TargetScan database [2,18], which predicts binding sites of miRNA families on targets based on the context scores. Genes with either conserved or poorly conserved binding sites for a miRNA were defined as targeting genes of interest for the corresponding miRNA. For each of the genes with multiple transcripts, only one transcript was selected as the representative transcript. Let *G *and *M *denote the total number of genes and miRNAs, respectively. The miRNA-target gene information can be mathematically described in the *G*-by-*M *miRNA-target matrix **T**.

$$T=\left\{t_{gm}\right\}$$

where *g *= 1,..., *G*, *m *= 1,..., *M*, and *t_gm _*is an indicator function, or *t_gm _*= 1 if *m*^th ^miRNA *binds to g*^th ^gene as defined above, otherwise, *t_gm _*= 0. Putative competitive endogenous RNA (ceRNA) pairs, defined as pairs of genes sharing common predicted binding miRNAs (*i.e*., miRNA programs or miRPs) according to previous reports [9,11], correspond to non-zero elements in the putative ceRNA matrix P, or

$$P_{G\times G}=T\times T^{\prime }$$

where **T**' is the transpose of **T**. For each putative ceRNA pair, we calculated the number of miRNA program binding sites (#miRPBS) by summing the numbers of binding sites of all miRNAs belonging to the miRP on the pair of ceRNAs.

### Statistical analysis

The Pearson correlation coefficient was utilized to measure degree of coexpression between expression profiles of pair of ceRNAs. In order to identify the essential factors that affect ceRNA regulation, we compared distributions of correlation coefficients obtained from different sets of putative ceRNA pairs. Each set of ceRNA pairs was derived by varying the size of miRPs, number of miRNA binding sites, etc., all essential factors for determination of ceRNA pairs. The goodness-of-fit between two cumulative distribution functions (CDFs) was evaluated with the two-sample Kolmogorov-Smirnov test (K-S test). By measuring the K-S statistic as the maximum vertical distance of two CDFs, the K-S test statistically infers whether two sets of samples are drawn from the same distribution and thus provides a nonparametric measure for comparing two CDFs. The putative ceRNA pairs satisfying the optimal states of essential factors in the GBM dataset were selected and defined as the "optimal ceRNA pairs".

### Construction and visualization of ceRNA networks

To confer higher-order signaling properties among ceRNA pairs, we first identified optimal ceRNA pairs and then constructed the ceRNA networks for various cancer types. Nodes and edges in the network denote ceRNAs and co-expressions between ceRNA pairs, respectively. We utilized the open source software Cytoscape [32] for visualization of the network.

## Competing interests

The authors declare that they have no competing interests.

## Authors' contributions

All authors conceived the study together. YuC designed the analysis pipeline and carried out the data analysis. TH and YiC revised the study design. YuC drafted the manuscript. YuC, YiC, and EYC reviewed and edited the manuscript and all authors read and approved the final manuscript.

## Supplementary Material

Additional file 1 **Figure S1**. Histogram of size of miRNA programs for all putative ceRNA pairs. Click here for file

Additional file 2 **Figure S2**. Histogram of number of miRNA program binding sites for all putative ceRNA pairs. Click here for file

Additional file 3 **Figure S3**. Histogram of expression levels of miRNA programs for all putative ceRNA pairs. Click here for file

Additional file 4 **Table S1**. List of optimal ceRNA pairs and summary of optimal ceRNAs. Click here for file

Additional file 5 **Table S2**. Lists of core ceRNA pairs and core ceRNAs in four cancer types. Click here for file

Additional file 6 **Figure S4**. Subnetworks of core ceRNA pairs related to cluster 1 (protein transport, detailed list of GO terms in Table 3) in cancers. Click here for file

Additional file 7 **Figure S5**. Subnetworks of core ceRNA pairs related to cluster 4 (protein modification/ubiquitination, detailed list of GO terms in Table 3) in cancers. Click here for file

Additional file 8 **Figure S6**. Number of core ceRNAs versus number of core ceRNA pairs exhibiting significant positive correlation with the core ceRNA regulatory networks. Click here for file

## Declarations

The publication costs for this article were funded by the Ministry of Science and Technology, Taiwan (MOST 103-2320-B-002-065-MY3).

This article has been published as part of *BMC Genomics *Volume 16 Supplement 4, 2015: Selected articles from the IEEE International Workshop on Genomic Signal Processing and Statistics (GENSIPS) 2013. The full contents of the supplement are available online at http://www.biomedcentral.com/bmcgenomics/supplements/16/S4.
