# Supplementary figures and images for: Parameter optimization for constructing competing endogenous RNA regulatory network in glioblastoma multiforme and other cancers
Source: BMC Genomics. 2015 Apr 21;16(Suppl 4):S1. doi: 10.1186/1471-2164-16-S4-S1 (PMC4416191; doi:10.1186/1471-2164-16-S4-S1)

Figure S1

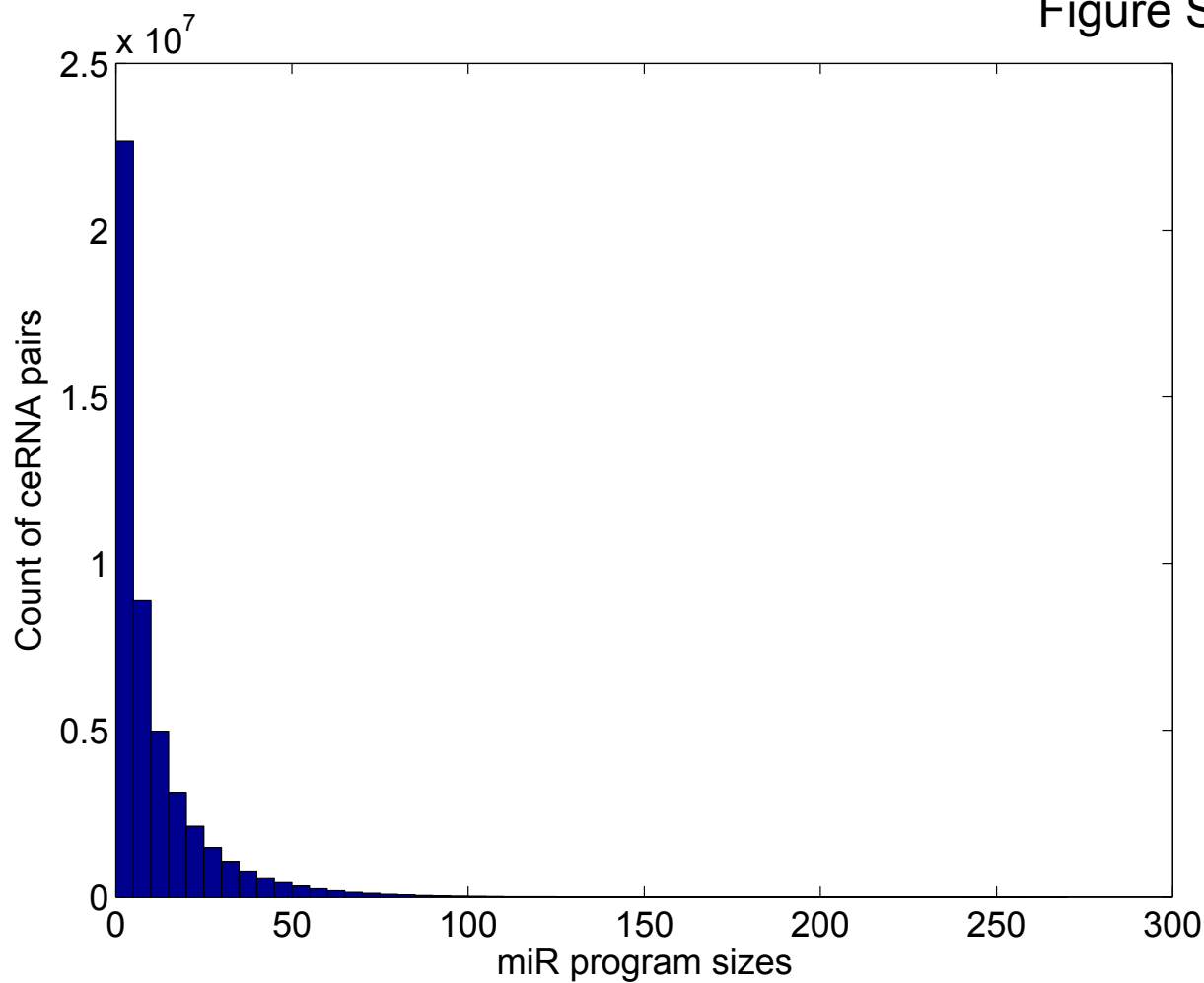

Supplement: Additional file 1 — Figure S1. Histogram of size of miRNA programs for all putative ceRNA pairs. [file 1471-2164-16-S4-S1-S1.pdf]

Figure S2

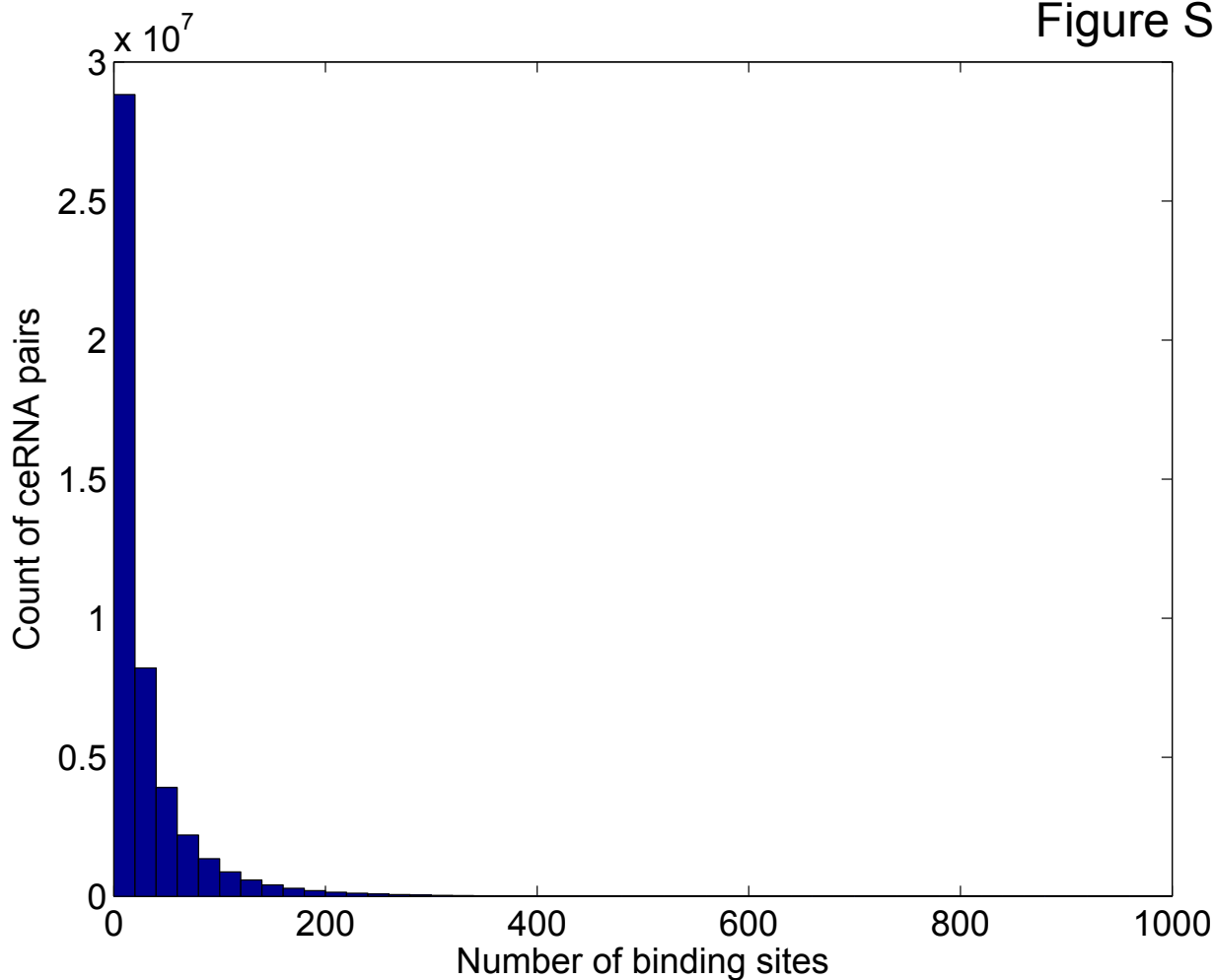

Supplement: Additional file 2 — Figure S2. Histogram of number of miRNA program binding sites for all putative ceRNA pairs. [file 1471-2164-16-S4-S1-S2.pdf]

Figure S3

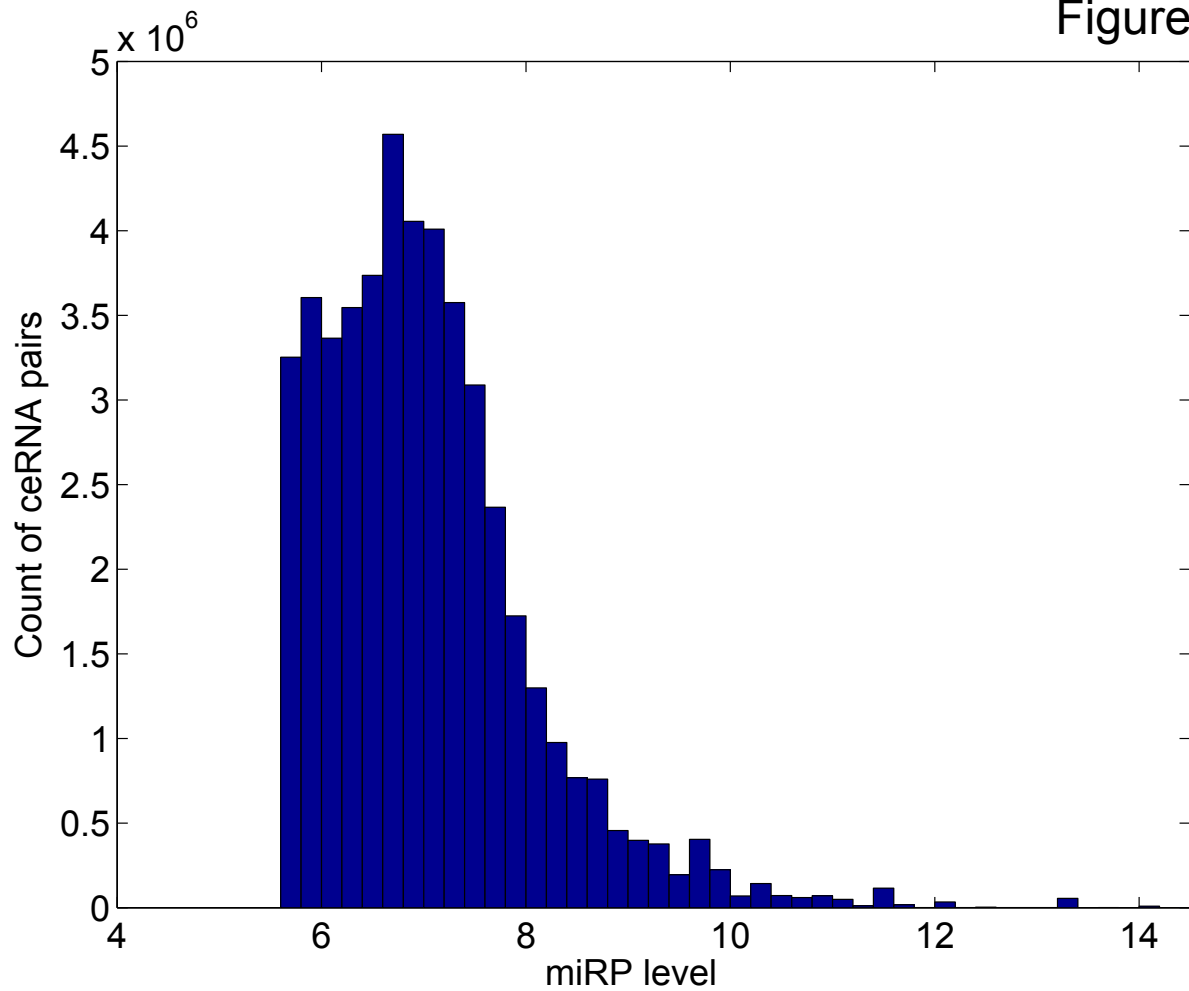

Supplement: Additional file 3 — Figure S3. Histogram of expression levels of miRNA programs for all putative ceRNA pairs. [file 1471-2164-16-S4-S1-S3.pdf]

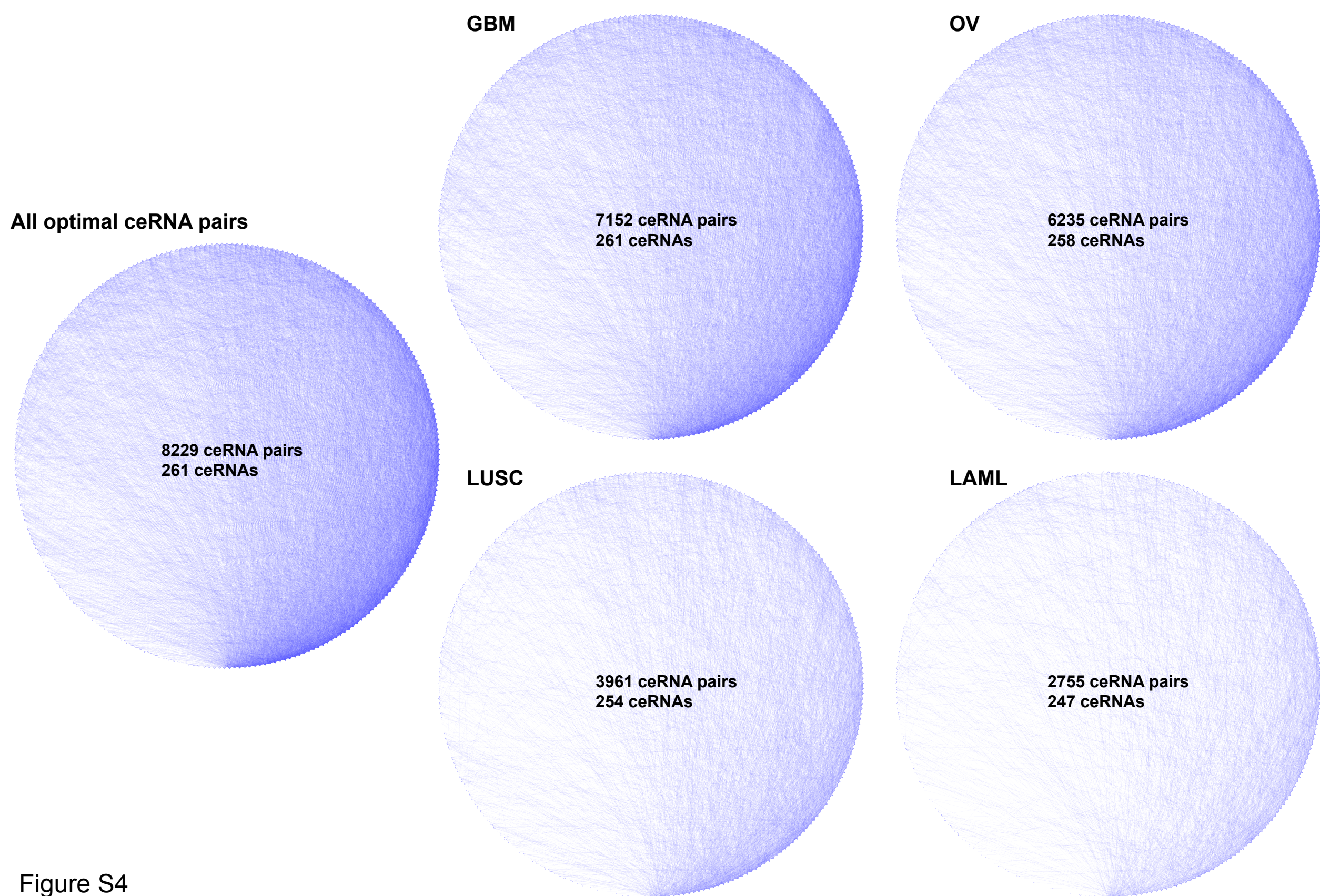

Figure S4

Supplement: Additional file 6 — Figure S4. Subnetworks of core ceRNA pairs related to cluster 1 (protein transport, detailed list of GO terms in Table 3) in cancers. [file 1471-2164-16-S4-S1-S6.pdf]

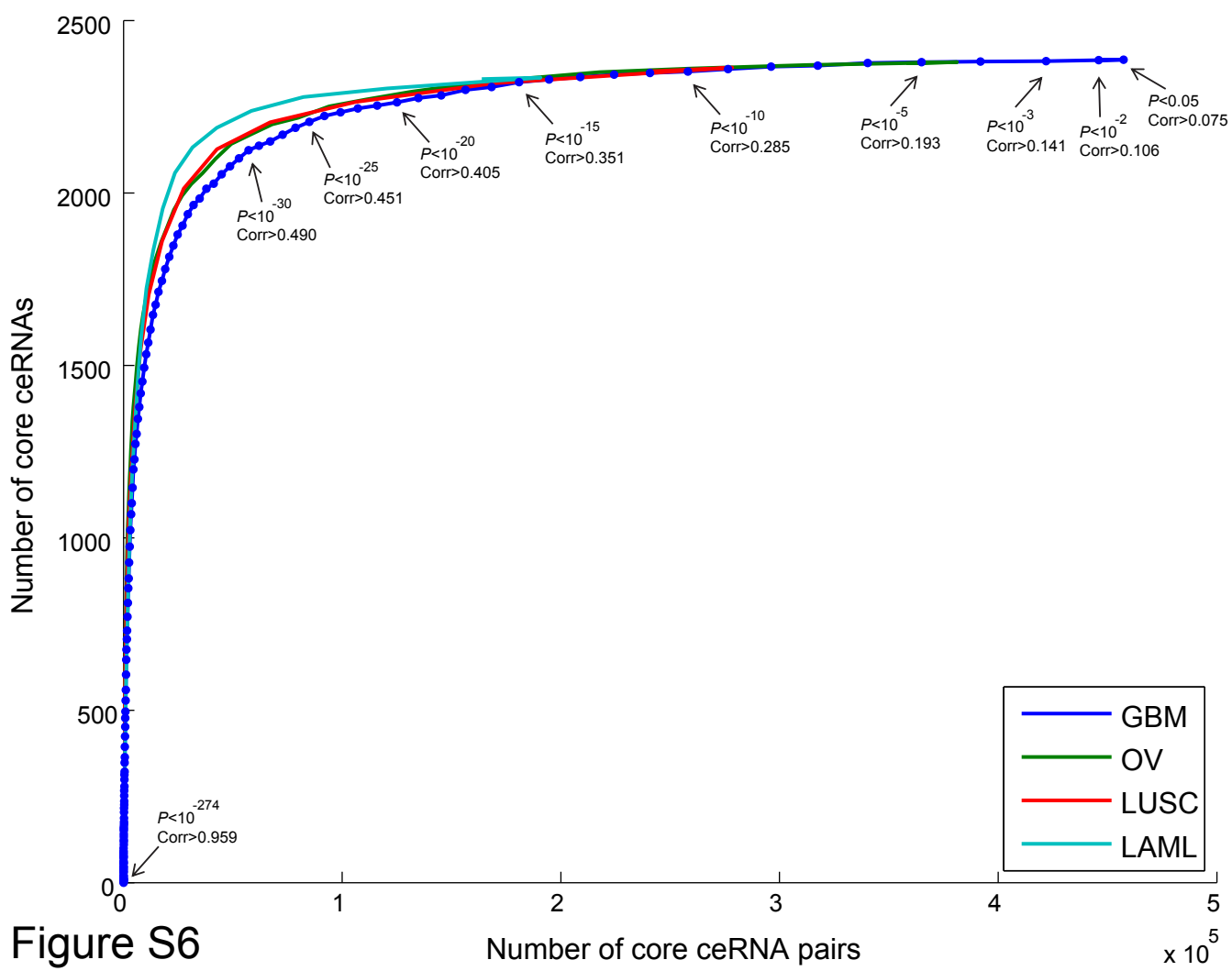

Figure S6

Supplement: Additional file 8 — Figure S6. Number of core ceRNAs versus number of core ceRNA pairs exhibiting significant positive correlation with the core ceRNA regulatory networks. [file 1471-2164-16-S4-S1-S8.pdf]
