# Supplementary material for: Parameter optimization for constructing competing endogenous RNA regulatory network in glioblastoma multiforme and other cancers
Source: BMC Genomics. 2015 Apr 21;16(Suppl 4):S1. doi: 10.1186/1471-2164-16-S4-S1 (PMC4416191; doi:10.1186/1471-2164-16-S4-S1)
Supplement: Additional file 7 — Figure S5. Subnetworks of core ceRNA pairs related to cluster 4 (protein modification/ubiquitination, detailed list of GO terms in Table 3) in cancers. [file 1471-2164-16-S4-S1-S7.pdf]

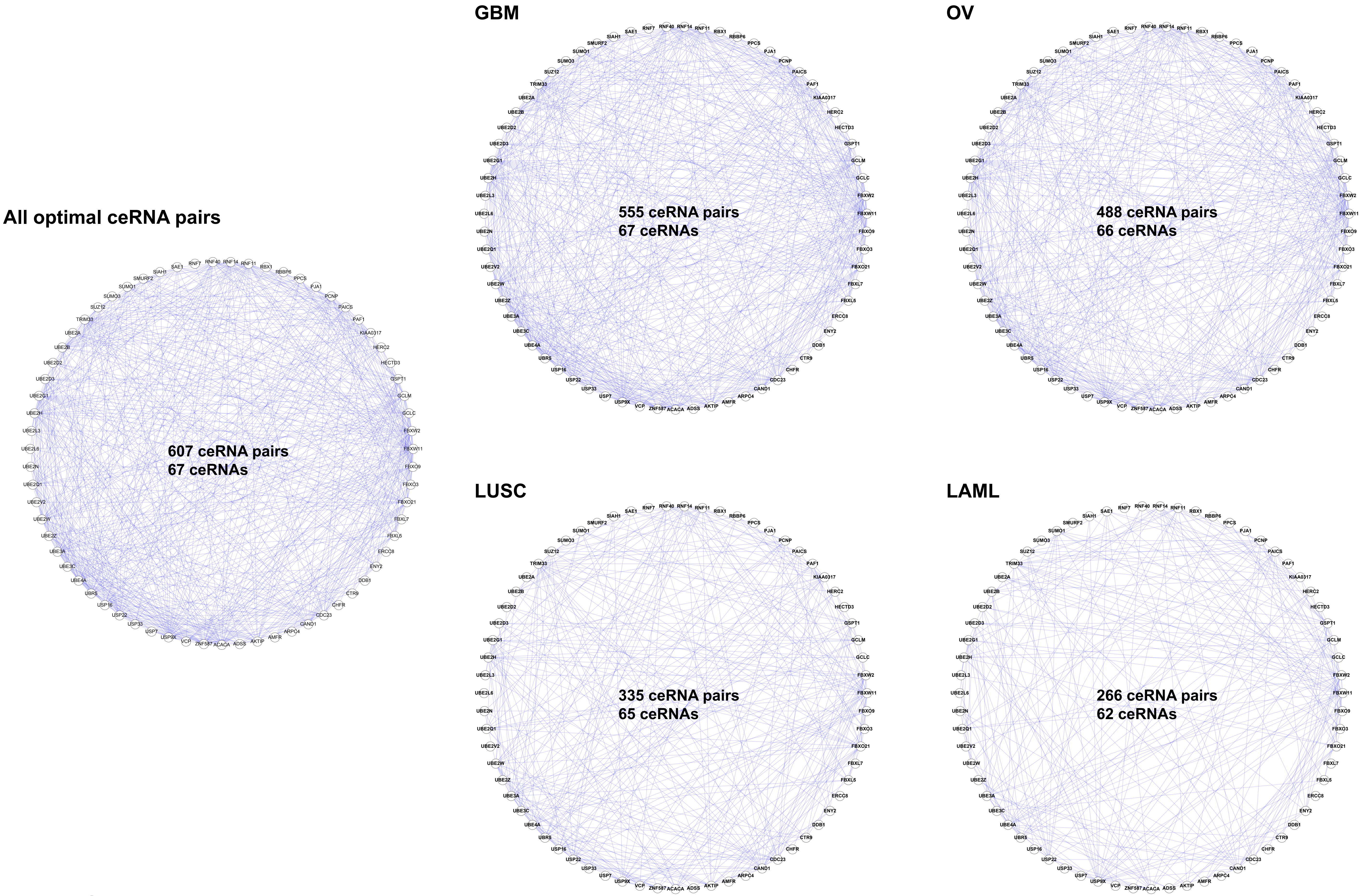
All optimal ceRNA pairs
607 ceRNA pairs
67 ceRNAs
GBM
555 ceRNA pairs
67 ceRNAs
OV
488 ceRNA pairs
66 ceRNAs
LUSC
335 ceRNA pairs
65 ceRNAs
LAML
266 ceRNA pairs
62 ceRNAs

Figure S5
